# Gamma power and beta envelope correlation are potential neural predictors of deep hypnosis

**DOI:** 10.1038/s41598-024-56633-x

**Published:** 2024-03-15

**Authors:** Yeganeh Farahzadi, Cameron Alldredge, Zoltán Kekecs

**Affiliations:** 1 Doctoral School of Psychology, ELTE Eötvös Loránd University, Budapest, 1064 Hungary; 2 Institute of Psychology, ELTE Eötvös Loránd University, Budapest, 1064 Hungary; 3 Department of Psychology and Neuroscience, Baylor University, Waco, TX 76798 USA

**Keywords:** Hypnosis, EEG, Machine learning, Interpretable AI, Psychology, Cognitive neuroscience, Consciousness

## Abstract

Hypnosis is a psychological intervention that is commonly used to enhance the effectiveness of therapeutic suggestions. Despite extensive fascination and study, the neural mechanisms behind hypnosis remain elusive. In the current study, we undertook a systematic exploration of these neural correlates. We first extracted well-studied neurophysiological features from EEG sensors and source-localized data using spectral analysis and two measures of functional connectivity: weighted phase lag index (wPLI) and power envelope correlation (PEC). Next, we developed classification models that predicted self-rated hypnotic experience based on the extracted feature sets. Our findings reveal that gamma power computed on sensor-level data and beta PEC computed between source-localized brain networks are the top predictors of hypnosis depth. Further, a SHapley Additive exPlanations (SHAP) analysis suggested reduced gamma power in the midline frontal area and increased beta PEC between interhemispheric Dorsal Attention Networks (DAN) contribute to the hypnotic experience. These results broaden our understanding of the neural correlates of deep hypnosis, highlighting potential targets for future research. Moreover, this study demonstrates the potential of using predictive models in understanding the neural underpinnings of self-reported hypnotic depth, offering a template for future investigations.

## Introduction

In this paper, we explore and investigate the neural correlates of what we refer to as “hypnotic depth”. The term “hypnosis” is subject to debate because it can encompass both a process and a mental state. The process of hypnosis involves a range of techniques designed to modify suggestibility and induce imaginative experiences^1,2^. Notably, based on the sociocognitive model of hypnosis^3^, an induction can be considered hypnosis, regardless of its surface characteristics, as long as it is accepted by the participant as hypnosis (i.e., it evokes the necessary expectancy, and it is presented in the right context). On the other hand, the hypnotic state is often described as a heightened focused attention, reduced peripheral awareness, and increased suggestibility^4^, although empirical evidence supporting improved focused attention and suggestibility following a hypnosis induction is limited^5^.

The experience of being in hypnosis is subjective and varies among individuals. However, some common phenomenological effects associated with a hypnotic experience include decreased peripheral attention^6^, deep relaxation, and vivid mental imagery^7^. The term “hypnotic depth” is used to describe the extent to which a person is immersed in this hypnotic experience^8^. The concept of ”depth” that implies significant changes in conscious experiences, is metaphorical^9^. However, the idea of consciousness having different depths is not merely a theoretical concept, but is also supported by empirical research showing a correlation between deeper levels and subjective and objective measurements^9^.

Despite the absence of a universally accepted standard for measuring hypnotic experiences, simple Likert style self-reported hypnotic depth scales (srHD)^10–12^ have shown high psychometric reliability^13^. These scales not only reflect the dimension of hypnotic depth but also correlate strongly with standard suggestion-based measures^10^, and comprehensive assessments like the Phenomenology of Consciousness Inventory - Hypnotic Assessment Procedure (PCI-HAP)^11,14^. In neuroscience of hypnosis, srHD scores have been effectively used, showing correlations with neural markers^8,15–17^ Also, in clinical settings, these scores are crucial as a client’s belief in their level of hypnosis is key to the success of hypnotic treatments^11^. In fact, self-reported hypnotic depth has been linked to the effectiveness of posthypnotic therapy^11^.

Furthermore, srHD scales offer a viable alternative to conventional hypnotizability tests. They are less influenced by suggestibility and are useful in measuring hypnotic depth in non-traditional settings^12^. This is significant, considering that hypnosis-like experiences can occur even without formal induction^4,18–20^, characterized by intense concentration^20,21^, mystical experiences^22^, and vivid imagination^7^.

In light of this background, our study aims to explore the neural correlates of subjective reports of deep hypnosis after different types of hypnotic inductions. We use four different induction methods, including both conventional and non-conventional approaches not previously explored in the existing literature. This inclusive approach is intended to broaden our understanding of the hypnotic phenomenon beyond the conventional procedural formality.

### Modulations in brain electrophysiological activity and brain intrinsic networks during hypnosis

It has been of special interest to study the modulations in brain electrophysiological activity and intrinsic brain networks associated with hypnotic experiences. Although studies in this area have not yet yielded a consistent picture of neural correlates of hypnosis, some promising patterns have been observed which will be discussed in this section.

Hypnosis has been linked to changes in specific neural oscillations, particularly theta and gamma waves, although these findings have not been consistently confirmed^23^. Prior evidence suggests that highly hypnotizable individuals (“highs”) demonstrate a higher baseline theta activity compared to those of low hypnotizability (“lows”) and that individuals, particularly highs, tend to show an increase in theta oscillatory power^23^ after a hypnotic induction. Moreover, the importance of theta waves in hypnosis is further highlighted by research showing that enhancing slow wave brain activity, achievable with practices such as neurofeedback and mindfulness meditation, may increase a person’s receptiveness to hypnotic interventions^24^. This suggests that brain theta wave activity could be instrumental in the effective processing of hypnotic suggestions, potentially improving a person’s responsiveness to such interventions.

There are, however, contradictory findings regarding the involvement of theta rhythms in hypnosis. For example, Cardeña et al.^17^ did not find any relationship between theta band power and level of hypnotizability or reported hypnosis depth. Additionally, a more recent study did not observe significant change in theta power from pre-hypnotic to post-hypnotic stages, challenging whether theta activity has a role in hypnotic experience^25^. The observed increase in theta activity could reflect a simple relaxation state^26^, or it may be due to the subject’s brain coupling with the speech rhythm (3–8 Hz)^27^, which is an integral part of most types of hypnosis inductions.

Research in hypnosis also pays great attention to gamma oscillations since they are thought to play a key role in various cognitive functions such as information processing, perception, attention, and memory. Gamma oscillations, particularly in terms of information processing, are thought to help integrate information across different brain regions, leading to cohesive perceptual and cognitive experiences^28^. Notably, neural activities within the gamma frequency range are often seen as a marker of local cortical activity^29,30^ and correlate most strongly with hemodynamic signals^31^. Therefore, changes in gamma power in specific brain areas are likely indicative of alterations in brain activity in those regions.

Accordingly, theories of hypnosis might imply that gamma oscillations should exhibit varying patterns of activity in relation to hypnosis and hypnotizability. Theories such as the cold control theory of hypnosis^32^ might indirectly imply reduced gamma power especially in the frontal area following hypnotic induction. This theory posits that hypnosis results from an individual’s intent to carry out a certain action, yet without conscious awareness of that intent, so reduced activity in those areas might be linked to a reduced ability to reflect on one’s own actions and intentions. An earlier theory by Woody and Bowers (1994)^33^ also predicted impaired frontal functioning during hypnosis. These theories are supported by findings that a disruption of activity in the left dorsolateral prefrontal cortex (DLPFC) increases hypnotic responsiveness, as evidenced in studies using repetitive transcranial magnetic stimulation (rTMS)^34^ (see also^35^ who did not see any effect after applying rTMS to the left DLPFC, but did find that right-sided stimulation of DLPFC increases hypnotic responsiveness). On the other hand, other theories emphasize the role of top-down processing through frontal networks, asserting that individuals who are highly hypnotizable can guide their attention to achieve more efficient and adaptable attentional control^36^. However, empirical findings on the direction of changes in gamma power following hypnotic induction is inconsistent, with some studies reporting *increase* in gamma power following an hypnotic induction^17^ while others reported a *decrease*^25,37^. This inconsistency may be due to different hypnotic induction techniques being used, the specific brain regions being examined, and the methods used to measure gamma power^23^.

Research using resting state fMRI provides complementary results; some evidence suggests that hypnosis is associated with changes in the activity of certain neural networks that are involved in modulating top-down attentional control and consciousness^38^. Previous research has demonstrated that a hypnotic induction may lead to decreased activity in the default mode network (DMN) while intra-network interactions of the salience network (SN) and the central executive network (CEN) increase in highly hypnotizable people following the induction phase^39^. However, a comprehensive meta-analysis of a wide range of studies, including those on hypnotic inductions and differnt kinds of suggestions, found that apart from the lingual gyrus, there was no consistent trend of activity changes in other brain regions in the reviewed neuroimaging studies^40^.

### Leveraging machine learning for reliable exploration of neural correlates in deep hypnosis

The reason that observed effects with significant p-values do not, in all cases, generalize to future observations may be partly due to the complexity and parameterization problems. Neural data is rich and complex, so statistical analysis and inference is not trivial. This is particularly evident in task-free studies exploring the impact of resting-state neural activity on cognitive and psychological traits^41^. Moreover, the variability in preprocessing and feature extraction methods further complicates the extraction of generalizable knowledge from neural data, highlighting the need for robust and reproducible approaches to discerning the neural underpinnings of hypnosis.

To tackle the complexity problem, machine learning algorithms, which adeptly handle high-dimensional data with minimal assumptions about the underlying stochastic processes, offer a promising solution^42^. In order to address the parameterization issue, it is often necessary to experiment with different feature subsets or to engage in feature engineering to identify the optimal set of features. Here, cross-validated grid search becomes instrumental. Thus, different feature sets can be treated as different hyperparameters, and the process of selecting the best set of features can be integrated into the hyperparameter tuning process. The feature sets can be included along with other hyperparameters, such as the regularization strength, in a grid of hyperparameters. Consequently, the optimal combination of hyperparameters, including the most suitable feature set, is determined based on performance metrics.

In light of these considerations, this study sets out to address this gap, applying best practices in machine learning to find neural correlates of deep hypnosis in order to produce reliable exploratory findings. Using the main neurophysiological measures referenced in hypnosis literature, we aim to develop interpretable models that will not only enhance current understanding but also lay the groundwork for future confirmatory studies.

## Methods

### Participants

52 participants (39 females, average age 24.5) were recruited from Eötvös Loránd University, School of Psychology. Inclusion criteria required participants to be over 18 years of age, healthy, and medically fit. Participants were excluded if they reported a history of epilepsy, schizophrenia, or other forms of psychosis or mental illness with symptoms of delusions or paranoia. Additionally, participants were not included if they had tried hypnosis previously or attended a hypnosis course. These eligibility criteria were assessed based on self-report.

Participants signed an informed consent prior to the experimental procedure and received vouchers worth approximately 25 USD as an appreciation for their time. Our study was approved by the Research Ethics Committee of the Faculty of Pedagogy and Psychology (Eötvös Loránd University, Budapest, Hungary; Ref. no.: 2021/345) and was conducted in accordance with the Declaration of Helsinki. Our data collection was also registered in a public trial registry, Open Science Framework on 30/03/2021, with the registration number: wvhda, and 10.17605/OSF.IO/WVHDA. However, the analysis presented in this paper is entirely exploratory, a scope which was explicitly outlined in our preregistration documentation.

### Procedures

**Figure 1:**
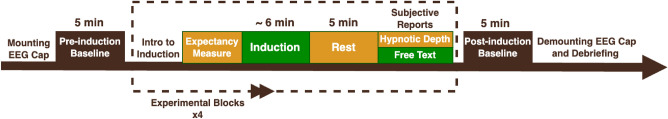
Experimental timeline: The present study is structured around four Experimental Blocks involving either conventional or unconventional hypnotic inductions, each described as either hypnosis or control techniques. The current study’s focus is to predict participant ratings of hypnosis depth, utilizing a variety of brain electrophysiological features extracted from five-minute Rest intervals, specifically during those Blocks in which the inductions were described as hypnosis. Data from the orange blocks is used in this current study.

As displayed in Fig. 1, after obtaining informed consent and mounting the EEG electrode cap, the study protocol started with 5 minutes of closed-eye rest (Pre-induction Baseline), followed by four experimental conditions (Experimental Blocks), and ended with another 5 minutes of closed-eyes rest (Post-induction Baseline). Throughout the four Experimental Blocks, participants were exposed to either conventional or unconventional (placebo) hypnotic inductions described either as hypnosis or as a control technique in a 2 x 2 balanced placebo design^43^. When a trial was described as control, participants were told that this is a control trial, and that brain imaging research and clinical research both indicate that this procedure can lead to relaxation but it does not produce a hypnotic state. In other words, each participant underwent four Experimental Blocks in which they were exposed to a conventional hypnotic induction presented as “hypnosis”, a conventional hypnotic induction presented as “control”, an unconventional hypnotic induction presented as “hypnosis”, and an unconventional hypnotic induction presented as “control”, all in a randomized order.

For conventional hypnosis induction, we used two commonly applied induction methods, relaxation induction^44^ and confusion induction techniques^45^. For the unconventional hypnosis, we used a so-called ”white noise hypnosis” procedure and ”embedded hypnosis”. ”White noise hypnosis” has been used in the previous studies consisting of white noise played to the participant described as hypnosis induction. In our study in trials where the technique was presented as hypnosis, participants were informed that subtle alterations in the frequencies of the white noise would induce a specific brain pattern associated with a hypnotic state. ”Embedded hypnosis” was specifically designed for this study. Participants listened to a story about the human body and muscles. In trials where the technique was presented as hypnosis, participants were told that subliminal suggestions were embedded in the audio on multiple volumes and frequencies, and that these messages were designed to remain unnoticed and only affect the unconscious mind. (see https://osf.io/pw5ye for a more detailed description).

In each experiment, participants began by reading a short description of the upcoming hypnotic technique. To check whether the manipulation worked, that is, that the procedure descriptions affected expectations about the soon-to-be-used technique, they were asked to rate their anticipated hypnotic depth on a scale ranging from 0 (Not Hypnotized at all) to 10 (Extremely Hypnotized).

In this study we treated all conditions as hypnosis conditions where the technique was described as hypnosis to the participant. This decision is grounded in the sociocognitive model of hypnosis^3^, which posits that any procedure accepted by the participant as hypnosis, regardless of its apparent characteristics, can be considered hypnosis as long as it evokes the necessary expectations. In our analysis we used the above-mentioned hypnosis depth expectancy ratings to ascertain that the expected hypnosis depth was comparable across the conditions that were described hypnosis regardless of the induction procedure used, and that the expected hypnosis depth was substantially lower in trials described as control (non-hypnotic).

After giving their expectancy rating, participants listened to a 6-minute induction recording. Post-induction, participants rested with their eyes closed for 5 minutes (”Rest”). A brief alerting signal then ended the Resting State. Afterwards, participants rated how ”deeply hypnotized” they felt during the Rest phase on an 11-point Likert scale ranging from 0 (not hypnotized) to 10 (extremely hypnotized).

We chose this straightforward Likert-like self-rating scale which is a valid method for self-reporting hypnotic depth, as detailed in the introduction. This approach was selected to enable participants to comfortably complete all four Experimental Blocks in a single session without experiencing fatigue. To gain deeper insights into their hypnotic experience, participants also provided written accounts detailing their sensations and feelings during the Rest phase.

Participants underwent these procedures while their electrophysiological activity was being recorded using Standard 128Ch BrainCap Sleep from Brain Products. In this study, 61 channels were used, including one ECG, two mastoids, and two EOGs electrodes.

In a second research session, we used The Harvard Group Scale of Hypnotic Susceptibility (HGSHS) to measure hypnotizability^46^ where 83% of the participants (43 individuals) completed this secondary evaluation.

### Analysis pipeline

**Figure 2:**
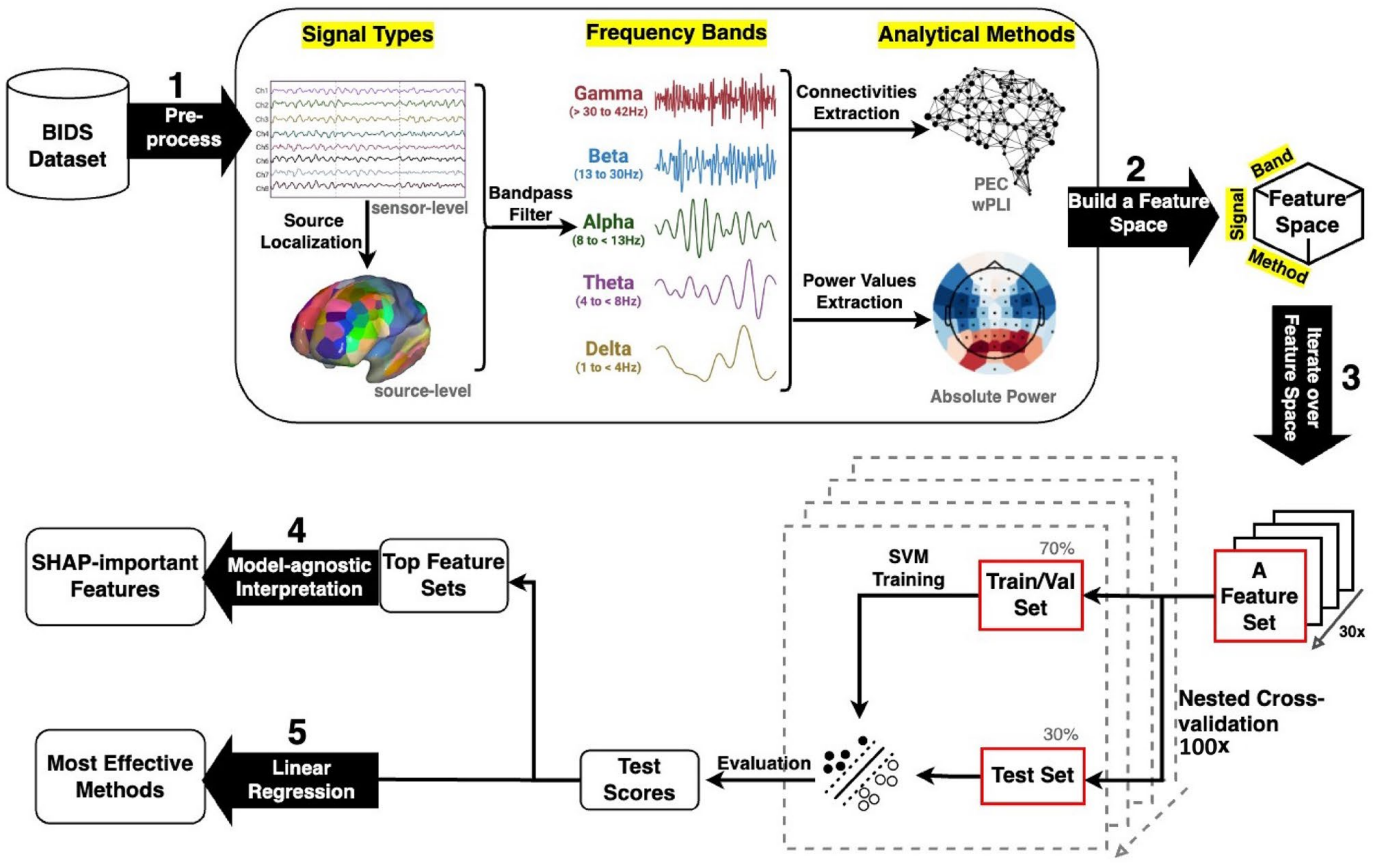
The analysis pipeline consists of five main steps: (1) Pre-processing: Initially, raw EEG data was preprocessed to correct for bad channels, remove transient and biological artifacts, and re-reference to the average of the electrodes. (2) Building a feature space: Then, the activity of seven brain networks were localized from the clean sensor-level data using an inverse model and Yeo et al. (2007) atlas. Subsequently, two connectivity measures (wPLI and PEC) and absolute power values were extracted from both sensor- and source-level data across five frequency bands resulting in a feature space comprising 30 feature sets. (3) Iterating over the feature space: The extracted features were used for a binary classification task (deep vs. superficial hypnosis depth). For each feature set, 70% of the data was allocated as the train set, while the remaining data was put aside as the test set. This test/train split was repeated 100 times, evaluating the model against the test set and generating 100 test scores. These scores were then used for (4) identifying the top two high-performing models and subsequently determining their most important features using SHapley Additive exPlanations (SHAP) values and (5) employing linear regression to determine the most effective analytical method based on the combination of features corresponding to each test score.

The analysis pipeline is shown in Fig. 2.

#### Data preprocessing

The following processing steps were applied to the EEG data using the python-based MNE package (v1.0.3)^47^.

- Extraction of each Experimental Block’s data from continuous raw data and organizing them into BIDS format using MNE-BIDS (v0.10)^48^.
- Visual detection of electrodes with poor signals and reconstruction using neighboring electrodes. At this step, we also visually inspected the data to detect the types of artifacts present. In total, 0.36% of all EEG channels across all participants were marked as bad.
- High-pass filter data at 1 Hz and stop-band at 42 Hz using zero-phase finite impulse response (FIR) filter with Hamming window.
- Removal of eye movement artifacts was performed separately for each Experimental Block by identifying and removing the eye movement components using MNE’s implementations of FastICA and CORRMAP^49^. Using CORRMAP, we manually selected three independent components representing typical blink and eye movement artifacts and used them as a template for selecting and excluding similar components for other participants.
- Epoching the continuous data into 1000 ms windows.
- Automatic rejection of remaining artifacts (head movements, transient jumps, drifts) using Autoreject v0.3.1^50^ and exclusion of epochs containing artifacts (7.8% of all the epochs removed at this step).
- Re-referencing to the average of the electrodes.
- Transforming the segmented data into a continuous format to prepare it for subsequent spectral and connectivity analysis.

Other than visually detecting bad channels, all other steps of preprocessing were automated, allowing reproducible results. In this automated pipeline, the dispersion vector^51^ was used to assess the quality of the data at the end of each preprocessing step.

### Feature extraction

#### Source reconstruction

Source reconstruction refers to the process of estimating the distribution of neural activity in the brain that gives rise to the scalp-recorded EEG signal. Although source reconstruction for EEG data without an individual T1 MRI from the subject is likely to be less accurate, this technique still allows for the investigation of brain activity at a finer spatial scale, which can be particularly useful when studying brain networks and their intra-connectivity^52^.

Source reconstruction is typically done using mathematical models that take into account the conductivity properties of the head, the location and orientation of the electrodes on the scalp, and the intrinsic spatial and temporal characteristics of the neural activity. The mathematical model we used was the exact low resolution electromagnetic tomography (eLORETA)^53^, which is suited for localization of brain resting state networks^52^. This method estimates the neural activity in each voxel (volume element) of the brain using a forward model that maps the scalp EEG data to the brain as well as a head model that specifies the geometry of the head and the conductivity of the scalp tissues. For the head model, we used MNE-Python’s template 3-layer boundary element method model. Template head models have been demonstrated to perform well compared to individual models derived from MRI^54^. The forward model assumes that the EEG data is generated by a combination of sources distributed throughout the brain. For all participants, we used the *fsaverage* surface template to compute forward operator from EEG data. Our source model was the canonical cortical surface implemented in MNE-Python consisting of 5,124 dipoles distributed along the cortical sheet. Dipoles were oriented normal to the surface. We used pre-induction baseline recordings to compute the covariance matrix.

The 5,124-dipole source space is then parcellated into 7 networks of interest^55^. These 7 networks - visual, somatomotor, dorsal attention, ventral attention, limbic, frontoparietal, and default networks - are thought to correspond to important functional systems in the brain, making the atlas useful for understanding the functional organization of the brain. We downsampled the data to 512 Hz before running source reconstruction.

#### Spectral analysis

**Figure 3:**
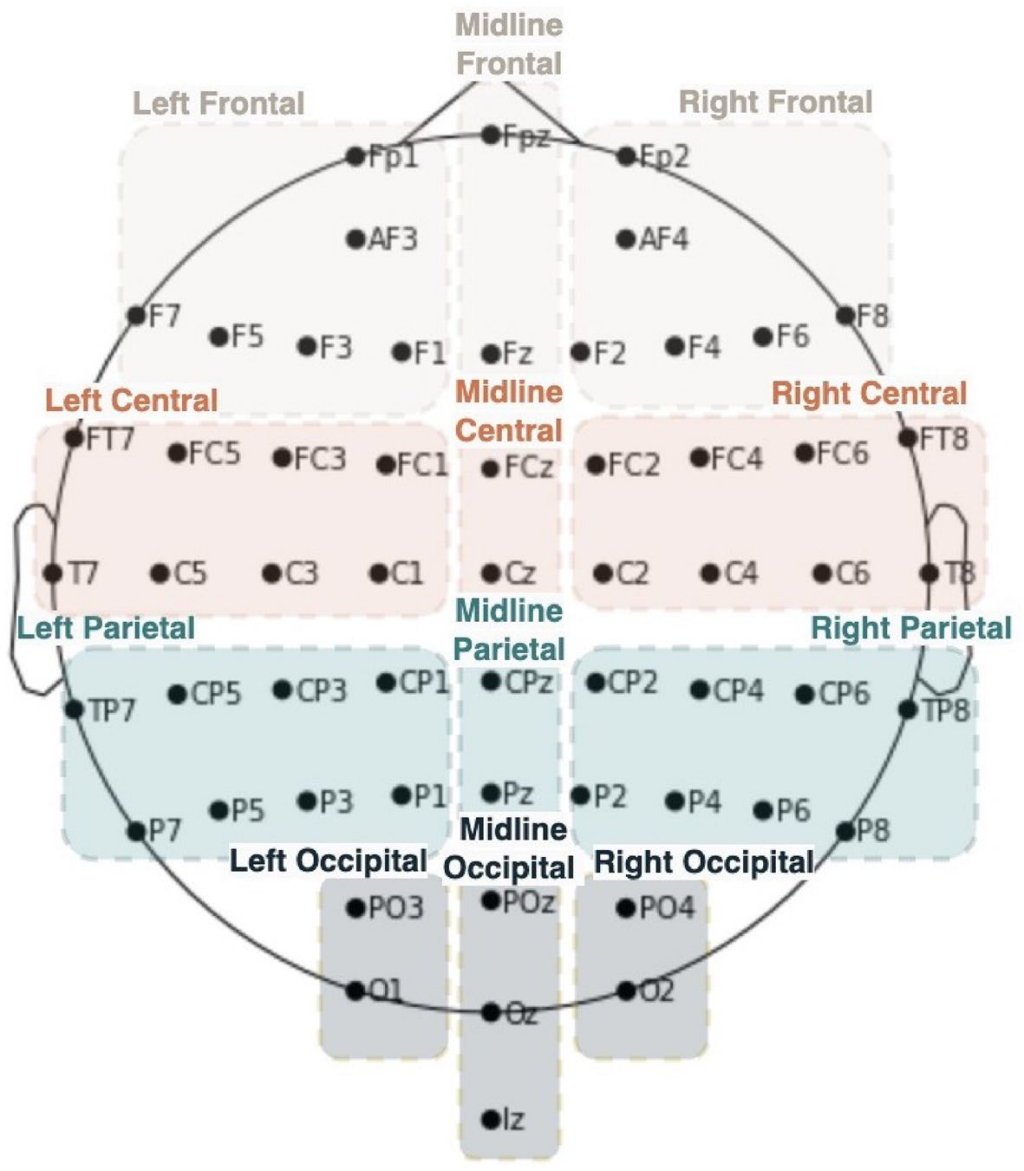
Electrode groupings.

Welch’s method was used to compute the power spectral density (PSD) for each subject at each Experimental Block (Hamming window length 8 s). We averaged each subject’s spectra over time within five standardized classical frequency bands (delta (1 to < 4 Hz), theta (4 to < 8 Hz), alpha (8 to < 13 Hz), beta (13 to 30 Hz), and low gamma (> 30 to 42 Hz) according to the Organization for Human Brain Mapping (OHBM) standards^56^ and then averaged the absolute power across all electrodes within each region of interest (Fig. 3).

#### Connectivity metrics

To construct a functional connectivity matrix during the resting state, we used two methods: weighted Phase Lag Index (wPLI), a measure of phase synchrony, and orthogonalized power envelope correlation (PEC), a measure of power synchrony. Both wPLI and PEC are robust and reliable measures of MEG/EEG functional connectivity estimation that eliminate spurious connectivity caused by limited spatial resolution of electrophysiological measurements^57,58^. Also, it has been shown that they can identify changes in brain functional connectivity in conditions associated with altered consciousness levels^59,60^. We calculated wPLI and PEC over time using the MNE-Connectivity (v0.5.0) package for both sensor and source-localized data. More specifically, we were interested in the changes in inter-network connectivity during hypnosis, since the inter-network crosstalk is considered to be essential for cognitive control and adaptive behaviors^61^. Before computing connectivity at sensor-level, we applied a spatial filter using spherical spline surface Laplacian transformation in order to minimize the volume conduction effects. Then, wPLI was calculated by taking the cross-spectral density between each pair of signals at each time point and frequency of interest using the Morlet wavelet. The number of cycles in the wavelet was set to be 5 cycles in each frequency band. For PEC, the complex spectral estimates were first calculated using the Hilbert transform on orthogonalized signals, followed by Pearson’s linear correlation calculated between the resulting power envelopes from two different places. The wPLI and PEC were calculated over time within a window of 30 s across all classical frequency bands, and were then averaged over all the time windows at each frequency to get the connectivity values for the entire 5-minute resting periods for each participant. This longer time window is suitable for EEG with lower sampling rate to capture slower dynamics of neural activity.

### Cross-validated grid search

The above analytical methods produced 30 different feature sets-3 analytical methods (spectral analysis, phase synchronization (wPLI), and power correlation (PEC)), 5 frequency bands (delta, theta, alpha, beta, and low gamma), and 2 signal types (sensor- or source-level data)-on each of which we trained separate classification models (Fig. 2). These classifier models received the extracted features from the brain for participants and predicted whether the person was in a deep or superficial hypnosis. Since Likert ratings are ordinal and shouldn’t be treated as real numbers^62^, we simplified our approach to a binary classification suitable for our sample size.) We selected the midpoint (5) as the cutoff, as it’s both physically meaningful for the scale and helps achieve a roughly equal number of samples per each class. In this case, hypnosis depth ratings equal to or less than 5 were considered superficial hypnosis while ratings above 5 were considered deep hypnosis.

We further conducted a sensitivity analysis using cutoff points of 4, 5, and 6 to examine how our choice of cutoff might affect the accuracy of different datasets. The details of this analysis are available in the supplementary materials S1. We also used participants' free-response self-reports to predict the classes of hypnotic depth (low vs. high) . Our aim was to assess whether  participants' numerical ratings are reliable and representative of the free-response reports  they provided. Using the self-report texts directly, without any additional feature engineering, resulted in a predictive accuracy of 68%. This results supports the use of numerical ratings as target variables in our main models (see supplementary materials S1 for more details).

Our classification pipeline included standardization, removing zero variances, and a Support Vector Machine Classifier (SVC) with linear kernel function which works well in high-dimensional spaces and is still effective in cases where there are more dimensions than samples^63^. To fine-tune the regularization parameter C of the SVC, we used GridSearchCV, testing a range of C values including 0.001, 0.01, 0.1, 1, and 10. The efficacy of each hyperparameter configuration was assessed based on accuracy as the evaluation metric.

We used a nested cross-validation approach within GridSearchCV. The outer loop of train-test split was randomized and repeated 100 times to get an average and a standard deviation of the prediction accuracies, an approach recommended for limited sample sizes^64^. In each split, 70% of the data was used to train and validate the model and the other 30% was used to test it. In the inner loop, which was the cross-validation within GridSearchCV, the 70% training data was further divided. Specifically, 20% of this data was set aside for validation, a process that was randomized and executed 30 times. As there were two rows of observations for each participant in the datasets, the GroupShuffleSplit method was used for both inner and outer cross-validation to ensure the validation and test sets included data of different individuals than those used in the training set. The cross-validated grid search in this study was implemented using the scikit-learn module in Python (v.1.2.2)^65^.

To determine a baseline performance for each model, we repeated the classification process using shuffled data instead of the actual data. The shuffling was done manually using the “shuffle” method from Python’s *random* module. Shuffling and training were repeated 100 times in order to obtain an empirical *p*-value against the null hypothesis that features and targets are independent^66^.

In a complementary analysis, we employed the same set of brain-extracted features and classifier pipeline—but without nested CV and tuning the parameter C—to predict hypnotizability scores. Due to the smaller sample size for these scores, we couldn’t reserve a validation set for hyperparameter tuning. In this context, we also divided the hypnotizability scores into two categories, using 6 (the midpoint of HGSHS scale and the median within our data) as the cutoff point. This resulted in 61% of participants falling into the high hypnotizable group, while 39% were categorized as low hypnotizable.

Furthermore, to isolate the specific effects of hypnosis, distinct from individual differences, we conducted an additional analysis. Here, we calculated the difference by subtracting the values of EEG features observed under the hypnosis condition (where the procedure was presented as hypnosis) from those in the control (where non-conventional induction procedures were introduced as controls). These differential values were then used as inputs for our models, aimed at predicting the differences in hypnotic depth between these two conditions. In this case, we also categorized the variations in hypnotic depth into two groups using a median split which corresponded to a score of 4. To maintain consistency with our primary analysis, we used the same classification pipeline with the same cross-validation method in this supplementary analysis S1.

### Regression inference

In this study, we used a rigorous approach which included testing multiple analytical methods on the sensor- and source-level data at different frequency bands. To assess which analytical method was most effective in terms of classification accuracy, we used a linear regression model with the $y \sim A * S + F$ formula. Where *y* represents prediction accuracy (in percent), *A* is a categorical variable representing the choice of analytical method (three levels including power spectral analysis, wPLI, and PEC measures of connectivity), *S* is a categorical variable with two levels indicating whether sensor- or source-level data was used, and *F* is a categorical variable representing the choice of frequency band (five levels). By including the interaction between signal type and analytical method, this linear regression model provides information on the performance of each method for sensor- or source-localized data, while also considering the main effects of the frequency bands.

We used test scores from the nested cross-validation pipeline described above. The 100-repeated train-test splits for each of the 30 different feature sets resulted in 3000 observations in total (3000 data points for *y*). The above linear model was aimed to fit this data of test score accuracy from repeated nested cross-validations. To fit this model to data, we used statsmodels module in Python (v0.14.0).

### Model diagnosis with SHAP values

The technical objective of this analysis was to interpret the contribution of individual features in the two top-performing feature sets, gamma power at sensor-level and beta PEC at source-localized data. These two feature sets also remained reliable across different thresholds for binarizing hypnotic depth scores (as detailed in the supplementary materials S1).

To understand the contribution of individual features on our top models’ output, we employed the SHapley Additive exPlanations (SHAP) method^67^. The contribution is the difference between the predicted output for a data point of interest and the average predicted output over all possible subsets of features, weighted by the number of possible subsets that include or exclude the feature. This is computed by retraining the model for each subset of features that includes or excludes the feature of interest^68^.

Unlike methods such as *multi-pass permutation importance* that focuses on the impact of features on a model’s performance, SHAP values focus on understanding what features are responsible for the output of the model, irrespective of whether the prediction is correct or not; therefore, it provides a deeper understanding of models that produce suboptimal predictions, since it focuses on the output of the model rather than its performance. Additionally, SHAP breaks the correlation between features by considering the effects of all the other features and interactions between them, making it a suitable choice for datasets with correlated features. To calculate SHAP values, we fitted the classification model 40 times with StratifiedGroup 5-fold cross validation for the top two feature sets.

## Results

### Conventional and unconventional hypnosis are equally expected to be effective

A two-way ANOVA was performed to analyze the effect of the type of induction (conventional or unconventional) and the description (hypnosis or control) on the expectancy of the participant regarding the effectiveness of a given procedure. The two-way ANOVA confirmed that the expectancy ratings do not vary significantly between conventional and unconventional hypnosis inductions, *F*(1, 204) = 0.0420, *p* = 0.8378 with tiny effect size $\eta _{p}^2$ = 0.0002. On the other hand, as expected based on the sociocognitive theory of hypnosis, description type had a significant effect on the expectancy of the participants *F*(1, 204) = 109.3086, *p* < 0.001 with large effect size $\eta _{p}^2$ = 0.3484. Also, the interaction effect between these two factors was not significant *F*(1, 204) = 0.5348, *p* = 0.4654, $\eta _{p}^2$ = 0.0026.

The level of hypnosis depth was different across different induction types and description types. Descriptive statistics showed that in cases of conventional hypnosis, describing the induction as control reduces the level of hypnosis depth by 2.46 points on average, while hypnosis depth ratings were only 0.81 points lower in unconventional compared to conventional hypnosis when both were described as hypnosis (Table 1). Two-way ANOVA also showed that hypnosis depth varied significantly across two different conventional and unconventional induction types, *F*(1, 204) = 6.5706, *p* = 0.0111, and two description types, *F*(1, 204) = 44.6733, *p*<0.001, with non-significant interaction, *F*(1, 204) = 0.3036, *p* = 0.5822. However, the effect size is small for the induction type factor ($\eta _{p}^2$ = 0.0312), and the interaction between two factors ($\eta _{p}^2$ = 0.0015) while it is relatively large for the description type ($\eta _{p}^2$ = 0.1796). Given the significant impact of description types on subjective experience, as indicated by the large effect size, we also explored how these differences are reflected at the neurophysiological level. For more details on this analysis, please refer to the supplementary materials S1.

**Table 1:** Comparison of mean subjective ratings of hypnosis depth and expectancy across levels of induction types and description types.

	Hypnosis		Control	
	Depth	Expectancy	Depth	Expectancy
Conventional	5.71 (2.73)	6.70 (1.96)	3.25 (3.01)	2.92 (2.52)
Unconventional	4.90 (3.37)	6.42 (2.14)	2.0 (2.36)	3.23 (2.78)

SDs are presented in parentheses, N = 52.

**Figure 4:**
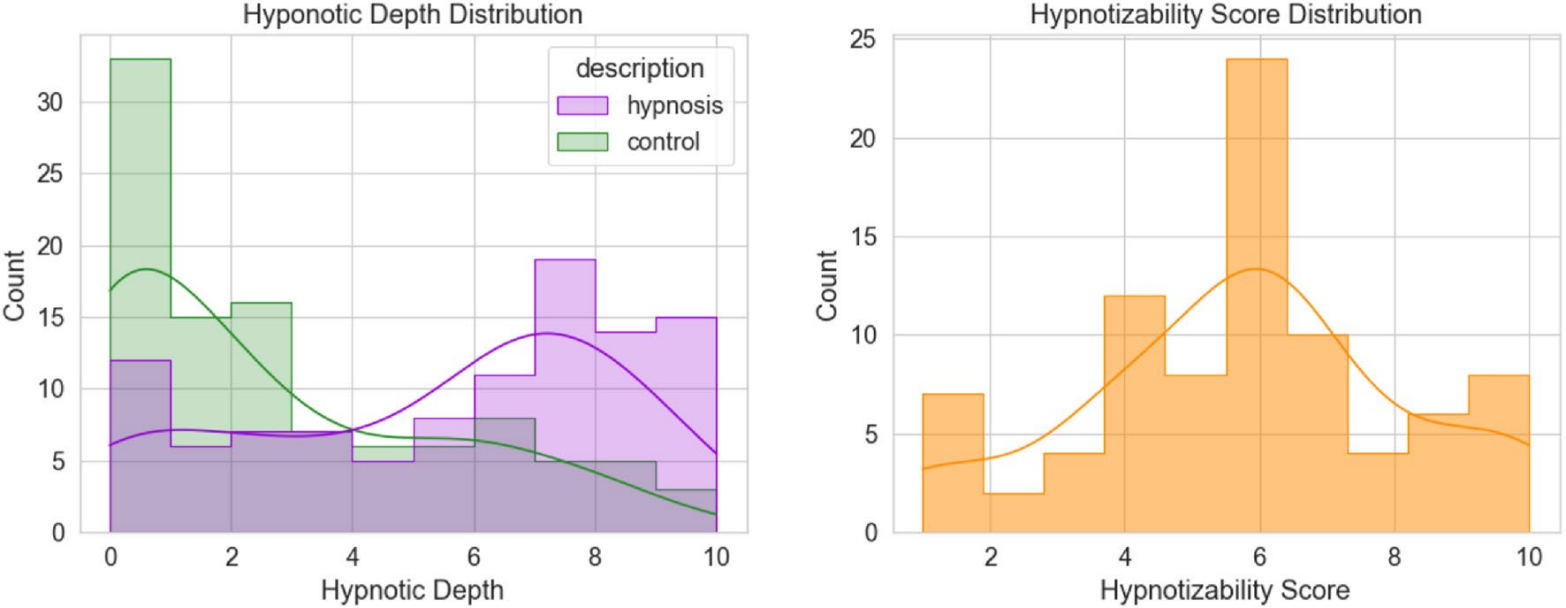
Hypnotic depth and hypnotizability score distributions. On the right distributions, the purple and green histograms show the distributions of the hypnotic depth ratings when the procedure was described as hypnosis and control respectively.

This result provides support for the sociocognitive model of hypnosis and indicates that regardless of whether a procedure is conventional hypnosis or not, the description of that procedure is the main determinant of the participants’ expectation of its effectiveness. Figure 4 shows the distribution of hypnotic depth ratings across participants for these two conditions. Consequently, in our study, both conventional and unconventional procedures presented as ”hypnosis” will be considered hypnosis conditions. Conversely, unconventional procedures presented as ”control” will be treated as control conditions. These conditions are especially appropriate to be considered as control, because not only they minimize participants’ hypnosis-specific expectations but also ensures a valid comparison with the hypnosis conditions due to their procedural similarities. Moreover, these conditions effectively counterbalance and eliminate potential order effects.

### Neural activities that involve faster oscillations might be counterproductive of hypnotic experience

**Figure 5:**
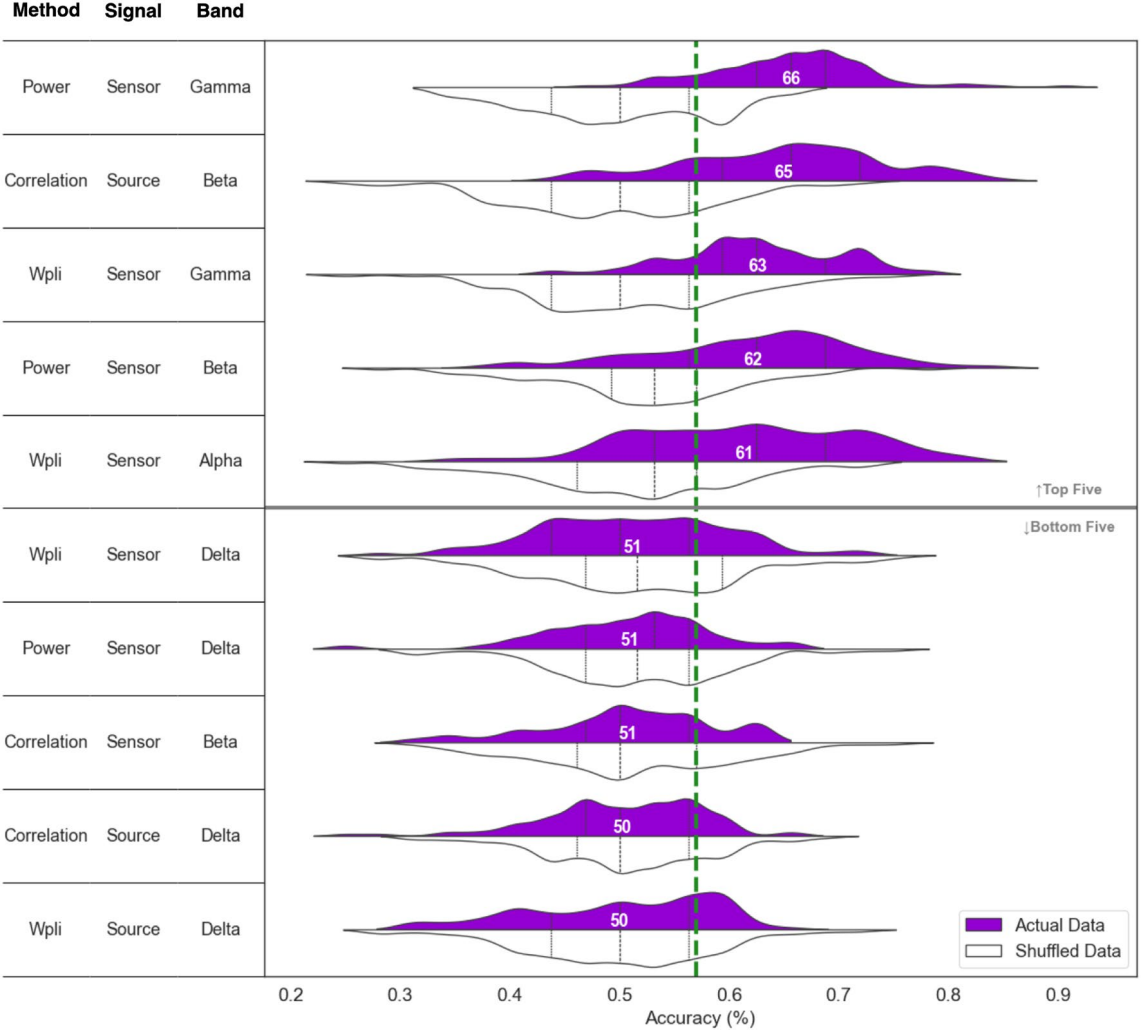
Performance of classifiers trained on different feature sets. This figure presents a comparison of the top five and bottom five classifiers based on their classification accuracies, ranked and annotated according to their average classification accuracies. The distributions in purple and white represent the accuracy spread of the models trained on actual data and permuted data, respectively. The dashed green vertical line marks the observed proportion of the dominant class (individuals with hypnotic depth ratings above 5) at 57%. For a complete view of the classifiers’ performance across all the feature sets, refer to the supplementary materials S1.

In unseen test sets, absolute gamma power at sensor-level and beta PEC at source-level showed the highest accuracy in classifying individuals into either deep or superficial hypnosis. Specifically, accuracy rates were 66 ± 0.073% for gamma power and 65 ± 0.091% for bata PEC (Fig. 5). Both models performed better than the chance level at 57% and also exceeded the performance of permuation-based null-models (*p* < 0.001; The average accuracy of the null models being 50.2% and 50% for gamma power and beta PEC respectively). However, while the model trained on gamma power showed high recall in accurately classifying the dominant class (deep hypnosis), showed less effectiveness in identifying the minority class (superficial hypnosis). Despite this, its performance still surpassed that of the permutation-based null model, as detailed in Table 2. In contrast, the model trained on beta PEC displayed a more balanced performance, with recall rates for both groups exceeding chance levels.

Notably, when we applied the extracted features to predict hypnotizability scores, beta PEC at source level exhibited the highest accuracy, achieving accuracies of 64%, and slightly surpassing the observed proportion of highs at 61%. (Please refer to the supplementary materials S1 for more details.).

**Table 2:** Comparison of recall, precision, and F1 score metrics between deep and superficial hypnosis groups for top models.

	Recall		Precision		F1 Score		Averaged F1 Score	
	Deep	Superficial	Deep	Superficial	Deep	Superficial	Weighted	Macro
Gamma power at Sensor	80, **65**	52, **31**	68, **56**	66, **40**	73, **58**	57, **33**	66, **48**	65, **46**
Beta PEC at source	69, **55**	62, **45**	70, **57**	61, **43**	68, **55**	60, **43**	65, **50**	64, **49**

The weighted and macro-averaged F1 scores are presented in the final two columns. Scores from the permutation-based null model are highlighted in bold for contrast. All values are reported as percentages.

Interestingly, when the differences in EEG features was utilized as inputs for our models, the gamma wPLI at the sensor level achieved the highest accuracy at 0.57± 0.10%, further supporting the importance of this feature set which was already implicated in the above-mentioned results. (For additional details, see the supplementary materials S1).

### Brain frontal area as well as control-related networks potentially contribute to classification accuracy

Using the SHAP analysis, we evaluated the importance of individual features on the classification output of the top two models, which includes gamma power at sensor-level and beta PEC at source-level. Here, the model’s output is the likelihood of assigning a given observation to one of two possible outcomes while accounting for other features and their interactions with each other.

**Figure 6:**
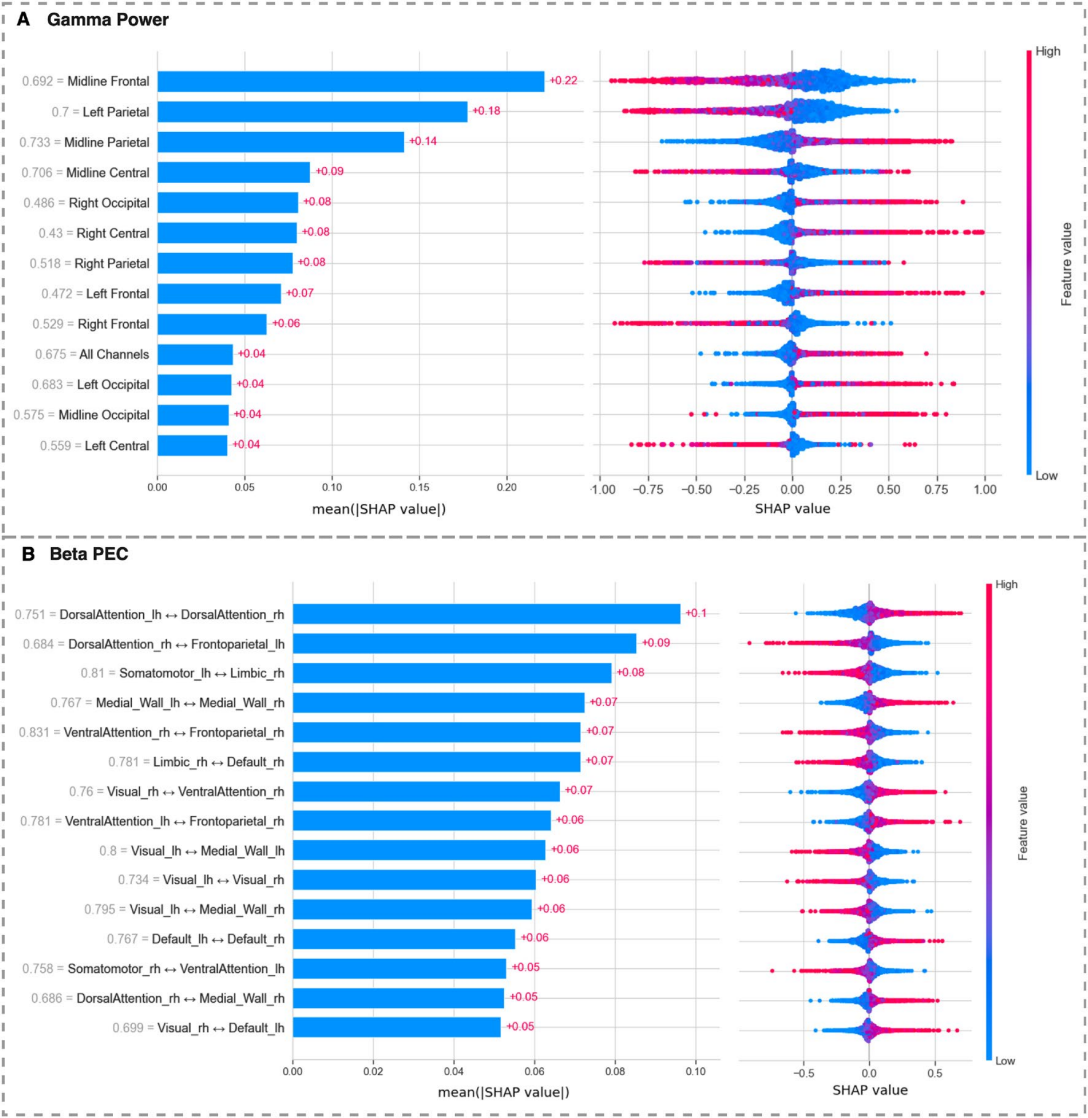
SHAP summary plots representing SHAP values across all data points. Each graph reads from top to bottom, showing the impact of each feature on the model output (superficial vs. deep hypnosis classification). Data are shown for (**A**) the absolute power from all the electrode groups at the gamma frequency and for (**B**) the top 15 inter-network power envelope correlations (PEC) at beta frequency band. The beeswarm plots on the right are composed of thousands of individual data points derived from several iterations within the training/testing phase, magnified by a 200$\times$ factor due to the model’s 40 repetitions and 5-fold cross-validation process. The color coding of these markers corresponds to the magnitude of the feature values, with warmer hues (reds) signifying higher values and cooler hues (blues) indicating lower values. This color scale is indicated by the ”feature value” bar on the right side of each plot. The position of the data points relative to the central line indicates the direction and strength of a feature’s impact on the model’s prediction. For example, in graph A, the accumulation of blue data points on the right side of the central line for features ’midline frontal’ and ’left parietal’ suggests that lower gamma power in these electrode groups is associated with positive SHAP values, therefore leading the model to move towards classifying the participant as ’deeply hypnotized’. On the bar plots to the left, the length of each bar reflects the average magnitude of the SHAP values ${\textbf {(mean(|SHAP|))}}$ calculated across all data points. These average values are marked in red on each bar. The gray numbers next to the names of each feature show the average of the absolute values of the features themselves.

The results of the top-performing feature set, gamma power, showed that the value in the midline frontal and left parietal areas had the highest impact on the classification output, with mean absolute SHAP values of 0.222 and 0.177, respectively (Fig. 6). The beeswarm graph for gamma power indicates that lower gamma power in midline frontal and left parietal areas is linked to a higher level of hypnosis depth, as the markers on right side of the central line are mainly blue, indicating an inverse association with the predicted level of hypnosis depth.

In the SHAP analysis of the model based on beta PEC, interhemispheric connections between the left and right dorsal attention networks (DAN) had a significant impact on the classification output (Fig. 6) with a mean absolute SHAP value of 0.096. The right-sided markers to the central line of beeswarm plot are mainly red in this case, indicating that higher interhemispheric connectivity between right and left DAN is associated with deeper hypnosis.

Other interesting internetwork connectivity that yielded significant results included the connections between control-related networks, dorsal attention network, and frontoparietal network (FPN). The lower connectivity between right DAN and left FPN (SHAP = 0.085) moved the hypnotic experience to a deeper level.

### wPLI slightly improves the accuracy, but not with source-localized data

We used a multiple linear regression model to investigate the effects of analytical methods (absolute power, wPLI, and PEC) and signal type (sensor-level and source-level) on accuracy in our sample of test scores. This model uses PEC connectivity in sensor-level data at alpha frequency as the baseline level. Our choice of reference here does not change the overall fit of the model or predictions made by the model. But it will change the way we interpret the coefficients so that we could only draw conclusion about the relationship between factor levels in relation to this reference category. Here, we remain on the descriptive report since we iterated over the same dataset, doing test/train splits. These descriptive results are summarized in Table 3 which shows that there were main effects for both analytical method and signal type.

For analytical methods, the coefficients for wPLI had a positive effect on accuracy $\beta \_wpli = 0.01$, which indicates that changing the analytical method to wPLI resulted in a 1% increase in test accuracy, holding all other variables constant. However, the interaction effect between wPLI and source ($\beta \_wpli*source = -0.04$) indicates that the effect of wPLI on accuracy varies depending on the type of signal being analyzed as wPLI will decrease the accuracy by 4% if it is used with source-localized data. There was a very subtle effect for power ($\beta \_power = 0.004$) while its interaction term with source had a slightly larger effect $\beta \_power*source = 0.02$.

For signal type, the coefficient for source was small ($\beta \_source = -0.01$), showing that source-level data had a slightly negative effect on accuracy compared to the baseline reference of PEC at the sensor-level.

However, the model statistics indicate a low level of explanatory power, with an adjusted R-squared value of 0.08, showing that only 8% of the variance in accuracy was explained by the predictors.

**Table 3:** Multiple linear regression descriptive results.

Predictors	Estimates
(Intercept)	0.57
Method [power]	− 0.004
Method [wpli]	0.01
Signal type [source]	− 0.01
Band [beta]	0.01
Band [delta]	− 0.04
Band [gamma]	0.02
Band [theta]	− 0.01
Method [power] $\times$ signal type [source]	0.02
Method [wpli] $\times$ signal type [source]	− 0.04

The table shows deflections of the accuracy from the baseline reference—which is PEC in sensor-level data at alpha frequency band.

## Discussion

In this study, we developed 30 different classifier models, each utilizing a unique combination of features extracted from neural electrophysiological activity data, to investigate the neural underpinnings associated with hypnotic experience. We developed 30 different models, each utilizing a unique combination of features extracted from neural electrophysiological activity data. By leveraging these computational techniques, we sought to uncover the patterns of brain activity that are most predictive of proposed hypnotic depth.

First, the combinations of different features that lead to the highest accuracy were gamma power at the sensor-level data and beta PEC at the source-localized data. This suggests that brain activity involving faster oscillations may be counterproductive for hypnotic experience.

Furthermore, a similar pattern emerged when we substituted hypnotic depth with hypnotizability scores in our models. The beta PEC feature set consistently stood out as the most significant. This consistency suggests a potential correlation between the simple hypnotic depth measurements used in our study and the established measures of hypnotic suggestibility. Therefore, the features we observed are likely reflective of the individual differences combined with the phenomenological changes induced by hypnosis.

Moreover, our research reveals that when differences in EEG features between hypnosis and control conditions are used as inputs for our models, the model employing gamma wPLI at the sensor level found to be particularly effective. This finding further emphasizes the importance of gamma oscillations in decoding the deep hypnosis experience. This is also in line with our primary analysis, which identified this feature set as the third most effective dataset. Indeed, gamma-band synchronized oscillations play a vital role in integrating information across sensory cortices, thereby greatly contributing to the formation of conscious experience^28^.

Our SHAP values analysis revealed that the most impactful features on the top model included reduced gamma power activity in the midline frontal and left parietal areas of the brain. The midline frontal electrode group consists of Fpz and Fz channels. Correlating EEG signals with exact cortical activities is challenging due to volume conduction. However, a simultaneous EEG-fMRI study^69^ showed that, particularly in frontal regions, there are minimal deviations between brain activities derived from EEG and those observed in fMRI scans. According to their observations, the Brodmann’s areas most closely associated with Fpz and Fz channels are BA10 and BA6^69^. BA10 is located in the anterior prefrontal cortex and encompasses the frontopolar cortex while BA6 is located in the precentral gyrus, just anterior to the primary motor cortex. These areas are involved in a variety of cognitive processes including control of attention and higher-order cognitive controls^70^. Since gamma oscillations may serve as a generic indicator of cortical activity^29,30^, the reduction of gamma power in these areas may reflect reduced functioning in the prefrontal areas. This finding is in line with previous studies showing that self reported level of hypnotic depth is correlated with reduced activity in anterior regions of the default model network^16^ especially the medial prefrontal cortex^8^.

Also, several hypnosis theories have hypothesized that a higher responsiveness to hypnotic suggestion may be associated with irregular frontal brain function^32–34,71,72^. This increased responsiveness to hypnosis is often associated with impaired executive monitoring^72^, or a reduction in rational thinking, and a tendency toward a more experiential and emotional perception of reality^71^. It can also occur due to inaccurate higher order thoughts regarding one’s intention^32^, which may be accompanied by impaired functioning of DLPFC brain regions associated with accurate higher order thoughts^34,35,73^.

Additionally, gamma oscillations are thought to be associated with the process of integrating and coordinating sensory information, which ultimately leads to the formation of perceptions of the physical world^28^. These oscillations have been observed to play a crucial role during various mental states, including hypnosis. As a result, theories of hypnosis emphasize this role by suggesting that successful responses to suggestions lead to a decrease in gamma activity in anterior areas of the brain, and possibly an increase in gamma power in the areas related to relaxing imagination^23^ However, the involvement and the specific changes in gamma oscillations during hypnosis are not universally consistent^23,74^.

Further, the SHAP values analysis on the beta PEC suggests that a stronger connection between left and right dorsal attention networks (DAN) is the most important feature for a high performing classifier that is correlated with deeper hypnosis. In fact, it is a fundamental characteristic of human brain anatomy that most homologous areas in both hemispheres are anatomically connected^75^. Accordingly, at rest, the left and right medial temporals, which are parts of the dorsal attention networks, show the strongest connections at beta frequency^58^. This suggests that the level of hypnotic experience seems to be pronounced through this basic characteristic of the brain during the resting state. Moreover, DAN plays a crucial role in our ability to selectively attend to relevant information in the environment and ignore irrelevant thoughts and information^76^. It is primarily involved in top-down, goal-directed attentional control, as opposed to bottom-up, stimulus-driven attention^77^. Consistent with this account and based on previous empirical findings^8,39^ hypnotic induction is thought to recruit those networks (including DAN) and its constituent brain regions to regulate attention and mental alertness^78^.

In addition, our analysis using SHAP values on beta PEC reveals the significant role of the other control-related network in hypnotic experience. We found that reduced inter-hemispheric connections between the frontoparietal network (FPN) and DAN is associated with more profound levels of hypnotic experience. Previous EEG studies also highlight the modulation of network connectivities within the frontoparietal network and the frontal-central and occipital areas (associated with DAN networks) following hypnotic induction^79,80^. These studies indicate that in individuals with high hypnotizability, there is a reduction in phase connectivities between frontal-parietal areas in the upper alpha band^79^ and the frontal-central and occipital areas in beta band^80^.

These observations based on SHAP analysis of beta PEC data suggest a potential increase in inward focus and a concurrent decrease in the processing of external information in individuals undergoing deeper levels of hypnosis. The altered connections between anterior and posterior areas also support dissociation and absorption theories of hypnosis^78^. However, the activity locations should not be over interpreted as the source localization was based on 59 EEG channels and without individual T1 MRI from participants.

Finally, in comparing test score accuracy obtained from different combinations of analytical methods and signal types, our findings suggest that both analytical method and signal type are important factors to consider when optimizing accuracy in modeling neural correlates of deep hypnosis. Specifically, wPLI was found to slightly increase accuracy compared to the baseline reference of PEC at the sensor level, while sensor signals were found to be slightly more informative than source signals, improving accuracy by around 1%. However, it is important to approach these findings with caution due to the small effect sizes and the post-hoc nature of this analysis.

The significant interaction effect between wPLI and signal type is particularly interesting, as it suggests that PEC may be more useful in this context for analyzing source-localized signals, compared to wPLI, which worked better on the sensor-level. This result is consistent with previous research suggesting that PEC is more effective than wPLI for analyzing source-localized EEG data to detect relative alterations in brain functional connectivity across different levels of consciousness^60^.

Our results also highlight the importance of considering the frequency band in analyzing neural signals, as higher frequency bands were found to be more informative for accuracy (Table 3). This may be due to the fact that the effect of drowsiness and mental fatigue is less evident on high frequency oscillations compared to low frequency oscillations. For instance, a recent systematic review showed that mental fatigue can induce the most significant changes and increase in theta activity in frontal, central, and posterior sites as well as moderate changes in alpha activity in central and posterior areas^81^. Considering the nature of our study, the low frequency oscillations might have a notably lower signal-to-noise ratio, possibly due to drowsiness. This could explain why models involving these slow oscillations were not among the top-performing ones.

It is crucial, however, to acknowledge that previous research has associated slow, especially theta, oscillations with the effects of hypnosis. Our study did not replicate these findings, possibly due to our distinct analytical approach. Prior studies compared brain activity changes between hypnosis and a pre-hypnosis baseline condition. In contrast, our research aimed to understand how deep hypnosis experiences is represented in the brain by analyzing the EEG signal characteristics directly within the hypnosis conditions. In other words, the observed differences in theta oscillation activities, when contrasting a baseline with a hypnosis condition, could be attributed to the influence of drowsiness, especially for low hypnotizable individuals, as these changes might naturally occur from pre- to post-hypnosis.

Our results are promising, but they also highlight areas for further exploration and refinement in future research. A key limitation of our study was the use of a simple unidimensional measure of hypnotic depth, chosen for its efficiency in allowing participants to complete four procedures in one session. While Likert-like self-report of hypnotic depth has its validity (see details in the introduction), previous studies indicate that there is a heterogeneity of subjective experiences in response to hypnosis induction. Also, phenomenological heterogeneity in response to an induction is very well established^82–84^. Thus, future research could benefit from incorporating a multidimensional measure to assess hypnotic experiences. One such tool is the *Phenomenology of Consciousness Inventory - Hypnotic Assessment Procedure (PCI-HAP)*^82^. This approach could provide a more nuanced understanding of the hypnotic experience.

Future studies could directly incorporate these rich free-response self-reports into predictive models. These models could potentially link brain-derived features, or even raw EEG signal values, with the content of these written self reports. An alternative approach might involve using broader categories or themes identified through sentiment analysis of the self-reports, using pre-trained language models that are fine-tuned for this specific dataset, or possibly employing sequence-to-sequence transformer-based models. Such predictive models, correlating EEG data with subjective experiences, hold significant promise and could yield important insights into the relationship between neural activity and personal perceptions. While the scope of these analytical possibilities is vast and exceeded the limits of our current study, we have made our data publicly available on the OpenNeuro platform. Interested researchers can access it through this link: https://openneuro.org/datasets/ds004572.

Additionally, our study predicted hypnotizability and hypnotic depth scores in two separate sets of models. Future research could explore the integration of these two aspects, potentially through a multi-head model that predicts both simultaneously. This model would categorize hypnotic depth while taking into account the individual’s level of hypnotizability. However, our sample size was not large enough to support the development of such a model, particularly as hypnotizability scores were available for only 83% of our participants.

Furthermore, it would also be interesting to investigate how hypnotic experiences may be related to the interaction of several features from different frequency bands rather than a single type of oscillation. Nevertheless, the sample size of the current study was not designed for such a huge feature space, and the computational capacity necessary to run such an analysis was not available for this project. With the aid of the results of the current study that illustrate how the individual feature sets contribute to the hypnotic experience, future studies may explore the interaction between these features to provide a more detailed understanding of hypnosis.

Lastly, this was an exploratory study in which we used a large variety of features and models. It is important to note that as an exploratory study, this research was not powered to detect the effect of any particular EEG feature, so features did performed poorly in our analysis should not be completely discarded as potential correlates of deep hypnosis. Furthermore, further feature engineering might enhance the importance of initially less significant features. Thus, a confirmatory study with a larger sample size and a more targeted approach could provide a more robust evaluation of the relationships between the variables. Overall, this study represents an important first step in this research area, and we hope that it will stimulate further research that builds upon our findings.

## Conclusion

In this study, we developed classification models that predicted self-rated hypnosis levels based on various high-dimensional features extracted from neural electrophysiological data. Our findings suggest brain activity involving faster oscillations may be counterproductive for hypnotic experience. Moreover, we found that subjective experience of hypnotic depth correlates with reduced gamma power in the midline frontal area and heightened interhemispheric connections between the left and right dorsal attention networks (DANs). This brain area and network are crucial for integrating information from various sources, cognitive control, and shifting attention between different stimuli.

Also, Our study utilized four different inductions, indicating that our results likely have broad applicability. This suggests that our findings are not limited to the specific induction methods employed, but instead reflect a general shift in how individuals perceive their depth of hypnosis. Our study also demonstrates the potential for using machine learning in research aiming to better understand the psychophysiology of hypnosis, and may serve as a template for future studies. Ultimately, a more comprehensive understanding of the neural correlates of deep hypnosis could have important implications for improving clinical hypnosis techniques and developing new therapeutic interventions for a range of psychological and medical conditions.

### Supplementary Information

Supplementary Information.

## Data Availability

The data used in this study is publicly available and can be accessed through the OpenNeuro Platform https://openneuro.org/datasets/ds004572. The codes used for analysis and modeling in this study are openly available and can be accessed on the project’s GitHub repository at https://github.com/Yeganehfrh/SugNet.
