# Supplementary material for: Gamma power and beta envelope correlation are potential neural predictors of deep hypnosis
Source: Sci Rep. 2024 Mar 15;14:6329. doi: 10.1038/s41598-024-56633-x (PMC10943225; doi:10.1038/s41598-024-56633-x)
Supplement: Supplementary file 1 — Supplementary Information. [file 41598_2024_56633_MOESM1_ESM.pdf]

Supplementary Information - Gamma Oscillations and Beta Envelope Correlation Are Potential Neural Predictors of Deep Hypnosis

Yeganeh Farahzadi[1, 2, *], **Cameron Alldredge**[3], **and Zoltán Kekecs**[2]

[1]Doctoral School of Psychology, ELTE Eötvös Loránd University, Budapest, 1064, Hungary
[2]Institute of Psychology, ELTE Eötvös Loránd University, Budapest, 1064, Hungary
[3]Department of Psychology and Neuroscience, Baylor University, Waco, TX76798, Texas, USA
[*]yeganeh.farahzadi@ppk.elte.hu

Text-Based Prediction of Hypnotic Depth

We trained a linear SVC to predict participants' hypnotic level (shallow vs. deep hypnosis) based on their free text reports to ensure numerical ratings are reliable measures of hypnosis depth. We used the "all-MiniLM-L6-v2" model from the embetter Python package to tokenize the text. On the validation set, the accuracy was 65%, significantly higher than the accuracy of the same model trained on the permuted data (Fig. 1). The GroupShuffleSplit method was used to perform cross validation 200 times with 80% of the data used for training and 20% for validation.

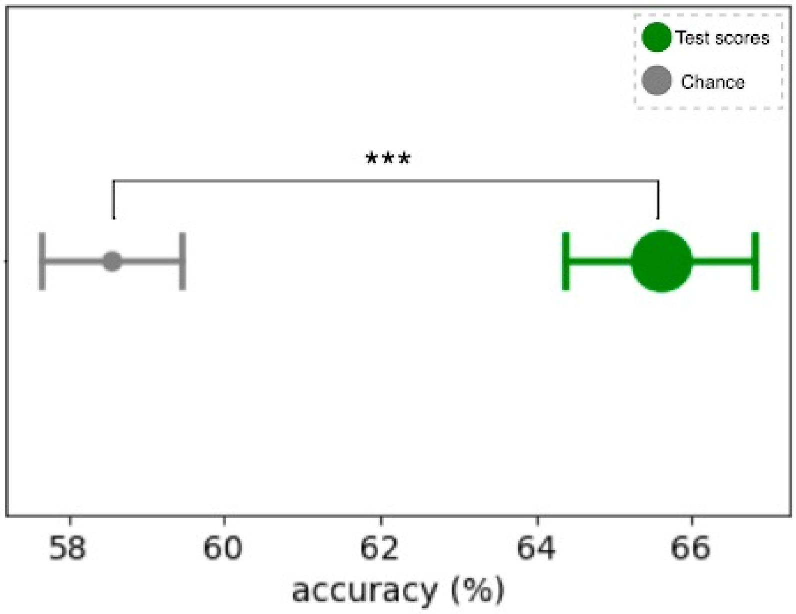

Figure 1. Deep vs. Shallow hypnosis classification as a function of embedded free text reports. The distribution of cross-validated out-of-sample prediction accuracy is displayed in green for the actual data and in gray for a shuffled version of the data (to form an empirical null distribution)

Predicting Hypnotizability Classes Based on Extracted Features from EEG data

In our supplementary analysis, we used the same brain-extracted features to predict levels of hypnotizability. Hypnotizability scores were divided into two groups using a cutoff of 6, representing both the HGSHS scale's midpoint and our dataset's median value. As a result, 61% of the participants were grouped as highly hypnotizable, and 39% as less hypnotizable. Similar to our primary analysis, we included only conditions introduced as hypnosis to participants. This meant we had two sets of EEG-derived features per participant. However, with only one hypnotizability score per participant, we duplicated this score for both sets of features. This duplication enabled us to maximize our data usage, but it also meant our hypnotizability data was somewhat limited. This limitation was exacerbated by the fact that 17% of participants did not attend the second session for hypnotizability measurement, reducing our data compared to hypnotic depth ratings.

Given the limited sample size for hypnotizability scores, we chose a non-nested cross-validation approach, which allowed more data in our training and testing sets. Consequently, our analysis did not include hyperparameter tuning. We employed the GroupShuffleSplit method for train/test splits, executed 200 times with repeated shuffling, using 70% of data as the train set and 30% as the test set in each split.

Figure 2 shows the accuracy of the top 10 datasets with the highest average accuracy. The model trained with beta PEC at the source level demonstrated the highest average accuracy at 63%, slightly above random chance. This finding suggests a potential correlation between hypnotizability and hypnotic depth scores, highlighting the importance of this feature in hypnosis research. However, the results should be interpreted cautiously due to the limited number of observations and the methodological constraint of using a single hypnotizability score in two rows of data.

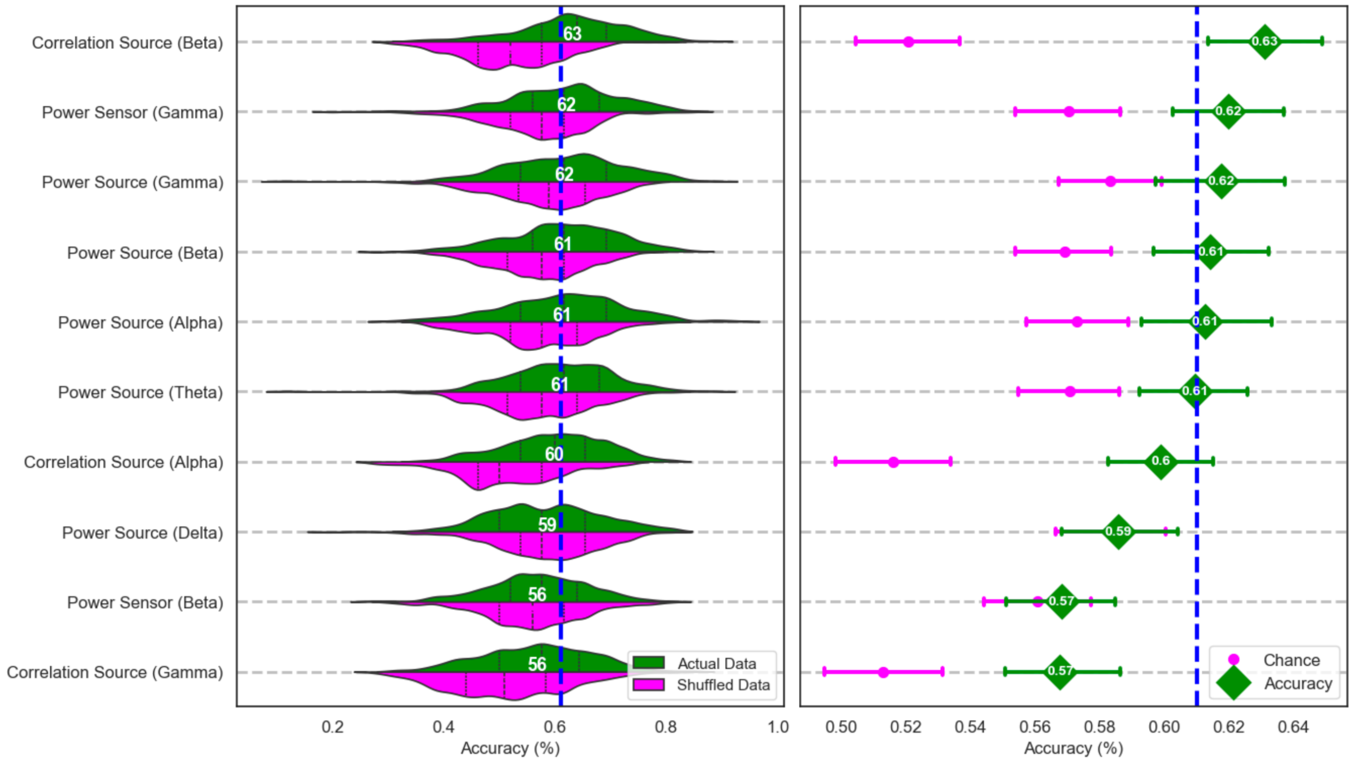

Figure 2. Top ten classifiers in predicting hypnotizability level. The green and purple distributions and points show the accuracies from the actual and permuted data, respectively. The dashed blue vertical lines indicates the true observed proportion of high hypnotizable group (chance level) at 61%. Error bars in the right graph represent 99% confidence intervals.

Sensitivity Analysis

In our study, we performed a sensitivity analysis to investigate how varying threshold levels, specifically 4, 5, and 6, affect our results. We utilized the same brain features for predicting hypnotic depth, grouping the data according to these thresholds. Our methodology did not include tuning the regularization parameter C, thus eliminating the need for nested cross-validation. For cross-validation, we employed the StratifiedGroupKfold method with a 5-fold approach, repeated 40 times to generate 200 data points for testing accuracy. Stratified cross-validation was essential due to significant differences in group proportions resulting from varying cutoffs.

Figure 3 presents the accuracy of each model across the three cutoff points. The models are ranked based on their average accuracy across these thresholds. This figure reveals that the top three models from our study consistently perform well, showing the least sensitivity to the chosen cutoff points. Specifically, the "gamma power at sensor" feature set consistently outperformed the null model regardless of the cutoff. This result highlights the robustness of our findings to threshold variations.

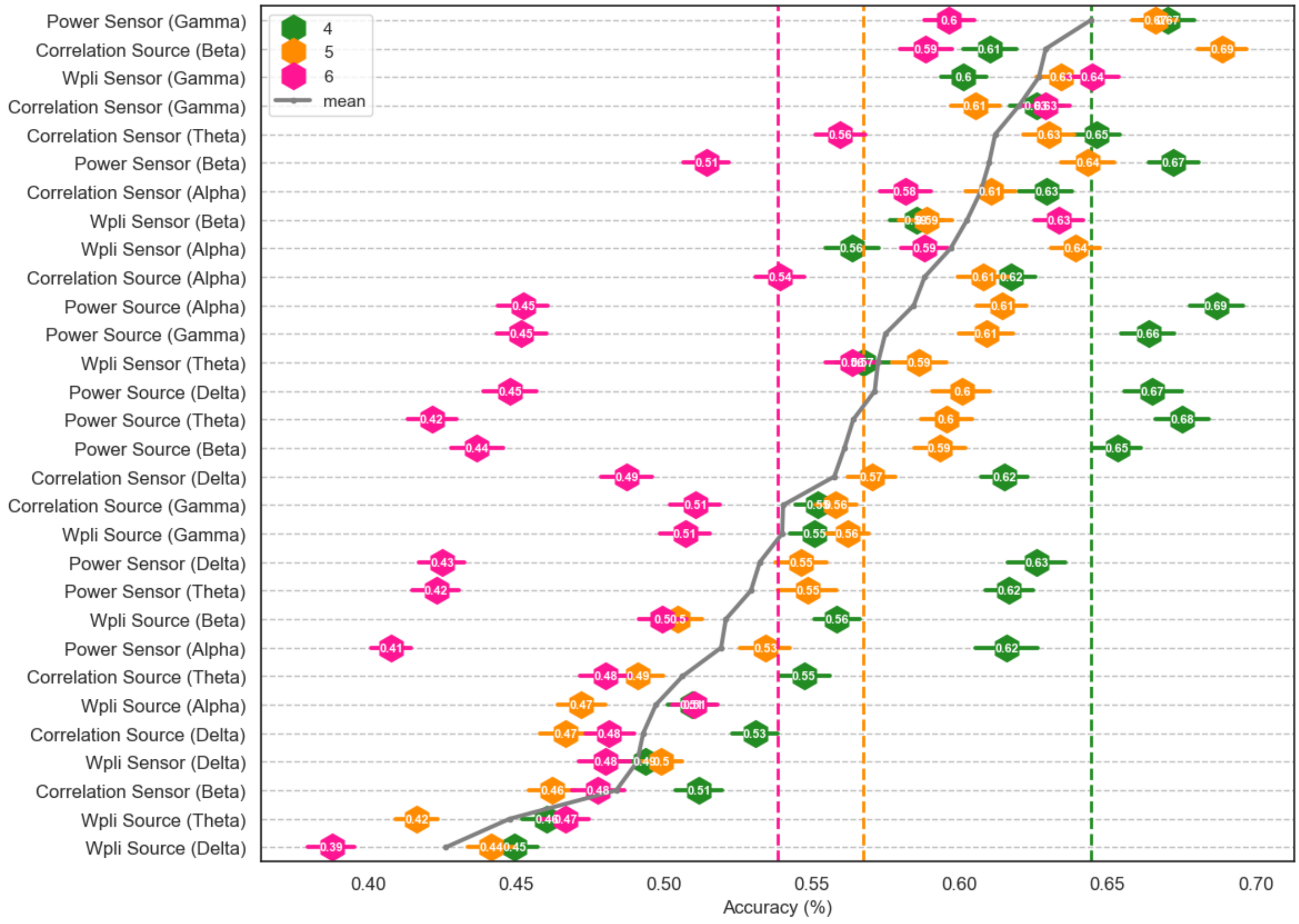

Figure 3. Impact of different cutoff thresholds on classification accuracy. The three vertical lines in green, orange, and pink represent respectively the baseline performance (chance level) when each of 4, 5, and 6 used as the cutoff points. The hexagonal markers with error bars indicating a 95% confidence interval are plotted in corresponding colors. These markers illustrate the classification accuracy achieved when using each of these cutoff points. The ranking of the models is based on the average accuracy (as indicated with the gray line) across three cutoffs

Predicting Description Types Based on EEG Features

This analysis focuses on predicting whether participants were told they were in a control or hypnosis condition during experimental trials. This prediction was based on features derived from EEG data. The data included four experimental trials with 52 participants. Due to a software error, we omitted data from one trial of one participant, leaving us with 207 observations for this analysis.

For this analysis, we used the same classification approach as in the main study. This involved using GridSearchCV to test and fine-tune the regularization parameter C for the model.

Figure 6 shows a ranking of different feature sets, ordered from most to least effective based on the average accuracy of the classifier trained using them. The findings indicate that the type of description given to participants (control or hypnosis) not only influenced their self-reported depth of hypnosis but was also evident in certain EEG feature sets. Notably, the beta Power Envelope Correlation (PEC) at the source level achieved the highest accuracy. This suggests that the beta PEC was effective in differentiating both the reported hypnotic depth and hypnotizability group of participants, as well as identifying the description type provided to them. This aligns with our observation, where the description given in each experimental trial significantly influenced participants' perception of their hypnotic depth.

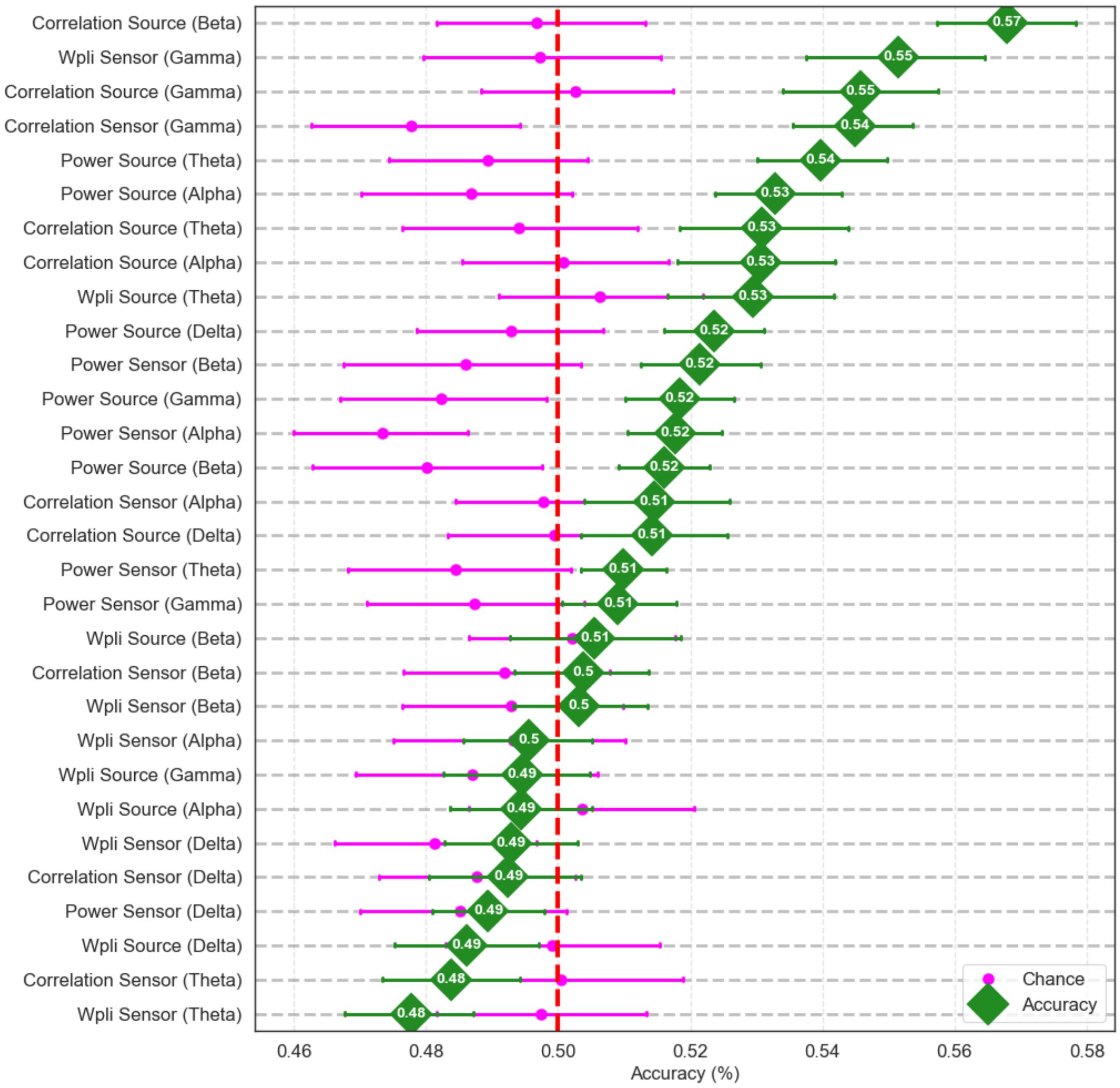

Figure 4. Description Type Classification based on brain Extracted Features. The red vertical line shows the chancel level at 50%. Error bars represent 99% confidence intervals.

Predicting Variations in Hypnotic Depth Based on Differences in EEG Features

This analysis focuses on predicting the variation in hypnotic depth rating based on the differences in EEG feature values between hypnosis and control conditions. Here, we used EEG feature values observed during the hypnosis conditions subtracted from those in the control condition as the inputs for our models. Similarly, in the model's output, we used hypnotic depth ratings in hypnosis conditions minus those in control, yielding a range from -8 to 10, with a median of 4. We categorized these hypnotic depth variations into two groups based on the median. As a result, 54% of participants were categorized as exhibiting greater differences induced by hypnosis, while 46% showed lesser changes from control to hypnosis intervention. To maintain consistency, we employed the same classification pipeline as in our primary analysis, which included using GridSearchCV for optimizing the regularization parameter C in our model.

Figure 5 presents the accuracy distributions and their corresponding confidence intervals for the top ten classifiers, ranked based on their average accuracy. Notably, only gamma wPLI (weighted phase lag index) connectivities at the sensor level showed significant performance above chance level with an accuracy of 0.57 ± 0.10. This result further underscores the previously suggested importance of gamma oscillations in our primary analysis. Such observations hint at a potential link between hypnosis and changes in gamma-band phase synchronization across different brain regions.

Moreover, our data show that 74% of participants who reported a more profound experience in hypnosis, as compared to the control condition, were those categorized as "high" in the hypnosis condition. This pattern suggests that individual differences in baseline gamma activity might play a role in these subjective experiences. Individuals might naturally differ in their baseline gamma levels, and these differences could be associated with their level of responsiveness to hypnotic induction. In essence, while our findings indicate an association between gamma-band activity and level of hypnotic depth, they also highlight the complexity of this relationship, influenced by individual neurophysiological profiles.

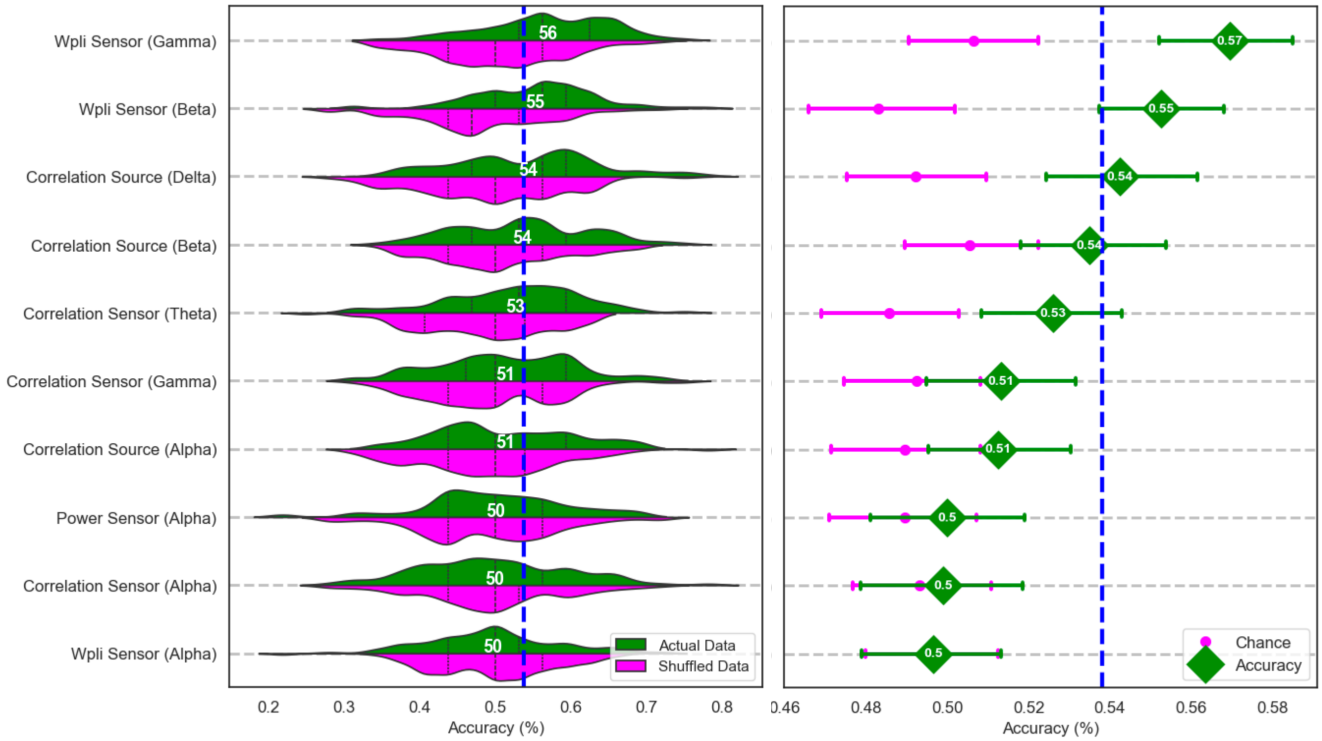

Figure 5. Top ten classifiers in predicting variations in hypnotic depth ratings from control to hypnosis conditions based on differences in EEG Features. The blue vertical lines shows the chancel levels at 54%. Error bars in the right graph represent 99% confidence intervals.

Comparison of the Classifiers' Performance Across All the Feature Sets

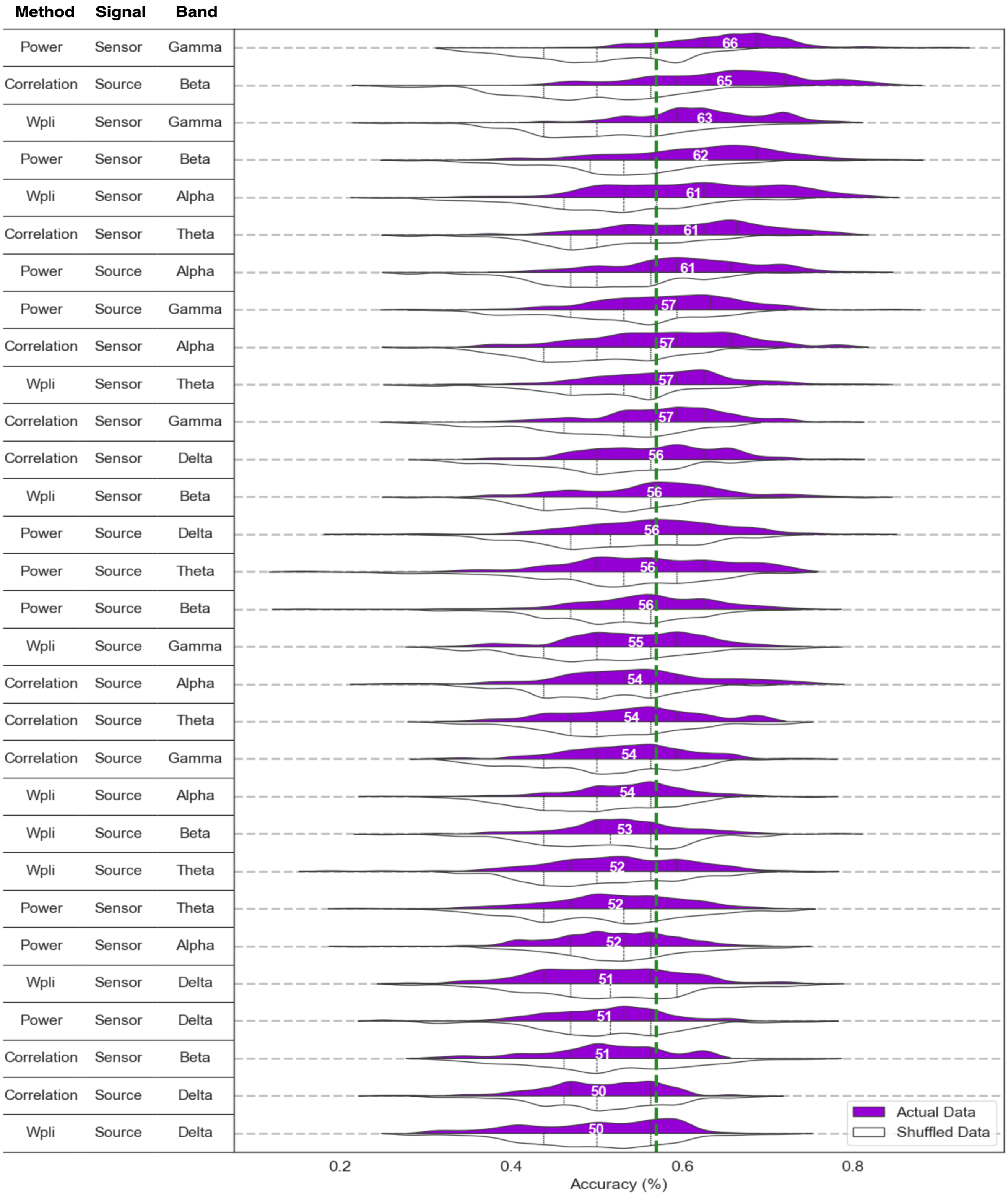

Figure 6. Classifiers' Accuracy Distributions for Actual and Permuted Data. The dashed green line indicates the chance level at 57%
